# Three-dimensional multi-parameter brain mapping using MR fingerprinting

**DOI:** 10.21203/rs.3.rs-2675278/v1

**Published:** 2023-03-24

**Authors:** Rajiv G. Menon, Azadeh Sharafi, Marco Muccio, Tyler Smith, Ilya Kister, Yulin Ge, Ravinder R. Regatte

**Affiliations:** New York University Grossman School of Medicine; Medical College of Wisconsin; New York University Grossman School of Medicine; New York University Grossman School of Medicine; New York University Grossman School of Medicine; New York University Grossman School of Medicine; New York University Grossman School of Medicine

**Keywords:** MR fingerprinting, brain imaging, quantitative mapping, multiple sclerosis, white matter lesion

## Abstract

The purpose of this study was to develop and test a 3D multi-parameter MR fingerprinting (MRF) method for brain imaging applications. The subject cohort included 5 healthy volunteers, repeatability tests done on 2 healthy volunteers and tested on two multiple sclerosis (MS) patients. A 3D-MRF imaging technique capable of quantifying T_1_, T_2_ and T_1ρ_ was used. The imaging sequence was tested in standardized phantoms and 3D-MRF brain imaging with multiple shots (1, 2 and 4) in healthy human volunteers and MS patients. Quantitative parametric maps for T_1_, T_2_, T_1ρ_, were generated. Mean gray matter (GM) and white matter (WM) ROIs were compared for each mapping technique, Bland-Altman plots and intra-class correlation coefficient (ICC) were used to assess repeatability and Student T-tests were used to compare results in MS patients. Standardized phantom studies demonstrated excellent agreement with reference T_1_/T_2/_T_1ρ_ mapping techniques. This study demonstrates that the 3D-MRF technique is able to simultaneously quantify T_1_, T_2_ and T_1ρ_ for tissue property characterization in a clinically feasible scan time. This multi-parametric approach offers increased potential to detect and differentiate brain lesions and to better test imaging biomarker hypotheses for several neurological diseases, including MS.

## Introduction

The utility of MRI mapping techniques quantifying voxel-wise relaxation times for multiple contrast mechanisms such as T_1_, T_2_, or T_1ρ_ have been shown in a number of neurological conditions ^1–4^. Despite their utility, they have not made their way to clinical protocols. A chief reason for this may be the typical extended scanning time needed to run the individual mapping sequences.

While T_1_ and T_2_ mapping have been developed decades earlier ^5,6^, T_1ρ_ contrast is a newer contrast mechanism which refers to the spin-lattice time constant in the rotating frame. It represents the decay of the transverse relaxation time in the presence of a spin-lock radiofrequency field ^7^. T_1ρ_ contrast has been shown to be sensitive to lower frequency range (KHz) interactions between free water and complex macromolecules ^8,9^. T_1ρ_ mapping has been widely implemented to quantify proteoglycan loss in cartilage ^9^, with clinical applications demonstrated in multiple sclerosis (MS) ^2,10^, Alzheimer’s ^11,12^ and stroke ^13^.

Newer, non-invasive multi-parameter MRI techniques ^14–16^ offering quantitative measures of key parameters can enable measurements to be collected within clinically feasible scan times. MR fingerprinting (MRF) offers a novel paradigm that allows simultaneous quantitative mapping of useful MRI parameters in a relatively short time ^17^. The utility of MRF has been shown in several pilot studies with applications in brain ^17–20^, heart ^21^, musculoskeletal and vascular applications ^22,23^.

MRF uses a different approach from conventional MRI, using a single imaging sequence to assess multiple tissue parameters such as T_1_ and T_2_, and therefore rapidly acquire fingerprint-like signal evolutions. A simulated dictionary is generated with combinations of possible signal evolutions. The acquired signal fingerprints are then pattern-matched to the pre-defined simulated dictionary to generate voxel-wise quantitative parameter maps for each parameter encoded into the acquisition. Current MRF implementations typically quantify T_1_ and T_2_ simultaneously. Based on a previous implementation, a 3D-MRF method capable of quantifying T_1_, T_2_ and T_1ρ_ was developed ^22,24^.

The goal of this study was to develop a 3D-MRF technique, capable of quantifying T_1_, T_2_ and T_1ρ_ for brain imaging applications, and to explore its feasibility for quantitative multi-parameter characterization in MS patients.

## Results

**Figure 1:**
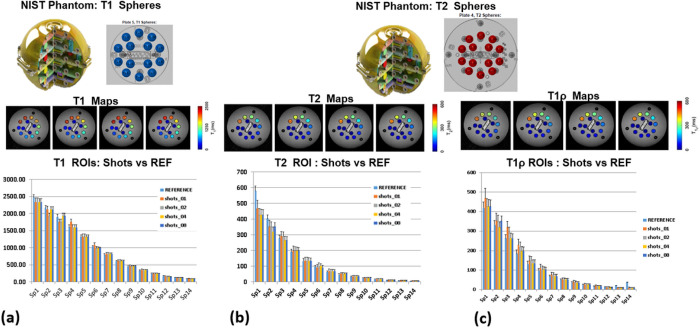
NIST phantom results tested with 1-, 2-, 4- and 8-shots. (a) shows the NIST phantom with T spheres, T maps for each shot tested, and the mean T_1_ values for each sphere reference compared to the 1-, 2-, 4- and 8-shots. (b) shows the NIST phantom T_2_ spheres, T_2_ maps for the shots tested, and comparison of shots to the reference, and (c) shows the T_1ρ_ maps for the shots tested, and their performance compared to reference.

Results from the phantom study are shown in figure 1. Figure 1(a) shows a picture and a schematic of the T_1_ spheres in the NIST phantom, the sub- figure below that shows the T_1_ maps reconstructed from 1-, 2-, 4- and 8-shots overlaid over the anatomical axial slice through the T_1_ spheres. The bar-graph at the bottom of Figure 1(a) shows the mean and standard deviation for each sphere comparing the reference method for T_1_, and the corresponding results from 1, 2, 4, and 8 shots. The range of values for T_1_ in the spheres was from 200–2500 ms, with the bar plot showing excellent agreement with the reference technique represented by a general trend of reduced standard deviation for each ROI with increasing number of shots. The T_2_ range was 0–600 ms, also showing similar excellent agreement. At the top of the range (>250 ms) there some variation of estimated T_2_ values between reference and multiple shots. The T_1ρ_ range was lower from 0–450 ms, with the estimated values at the higher values (>200 ms) showing greater variability among the shots and with the reference technique. Increasing number of shots reduces the variability in each ROI, with 4 and 8 shots showing the least variability. While there is great agreement even with one shot, variability in estimated parameters increase with lowest shots (1 shot) and at higher end of the ranges for T_1_, T_2_ and T_1ρ_.

**Figure 2:**
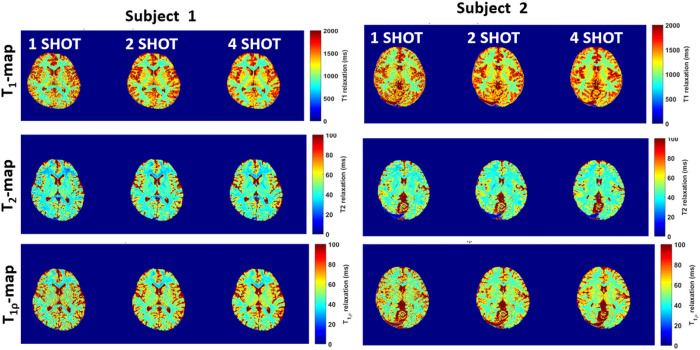
Comparison of shots in vivo. The figure shows exemplary T_1_, T_2_ and T_1ρ_ 1×1×3 mm^3^ resolution for 1, 2 and 4 shots. The SNR improves significantly from 1 to 4 shots, at the cost of increased acquisition time. Two shots deemed as a suitable balance between SNR and clinically feasible time was used for scanning MS patients.

Figure 2 shows a comparison of multi-parameter maps for 2 exemplary control subjects for 1-, 2- and 4-shots obtained using 3D-MRF, highlighting the ability of the technique to generate 3D, contiguous and simultaneous parameter maps for T_1_, T_2_ and T_1ρ_. The SNR using 1 shot was 6.45 ± 1.6, using 2 shots was 8.3 ± 1.9 and using 4 shots was 11.4 ± 2.1.

**Figure 3:**
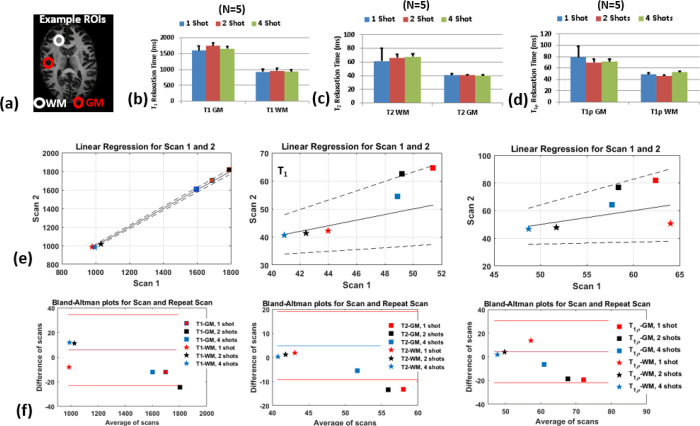
Comparison of multi-parameter maps with shots and ROIs. (a) shows WM and GM ROIs used. The performance of for GM and WM ROIs with 1, 2 and 4 shots are shown for (b) T1, (c) T2, and (d) T1ρ. (e) and (f) shows linear regression and Bland-Altman plots for repeatability from two separate scans are shown for T1, T2 and T1ρ

Figure 3(a) shows representative ROIs drawn in the GM and WM in healthy subjects. Figure 3(b) shows mean T_1_ values, figure 3(c) shows T_2_ values and figure 3(d) shows T_1ρ_ values obtained from the GM and WM ROIs in the control subjects with 1-, 2-, and 4-shots. The T_1_, T_2_ and T_1ρ_ values showed more variation in the GM compared to the WM, with the variability reducing with increased shots. The results from 2 shots were comparable to that of 4 shots. Table 1 summarizes the results from the control subjects using 1-, 2- and 4-shots.

Figure 3(e) and (f) shows the regression plots and Bland-Altman plots testing repeatability for T_1_ T_2_ and T_1ρ_ values, respectively. While the parameter values were in excellent agreement, the T_2_-GM values showed more variability compared the WM values. Results from 2- and 4-shots showed greater agreement than 1 shot. The results in Table 2 show the intra-class correlation coefficient (ICC) in the WM and GM ROIs for 1-, 2- and 4-shots. The results show that for 2- and 4-shots show the highest correlations. From these results, the 3D-MRF acquisition using 2 shots was considered optimal taking into consideration a clinically feasible imaging time (~12 min), SNR, noise characteristics, and accuracy, and was subsequently used for imaging in MS patients.

**Figure 4:**
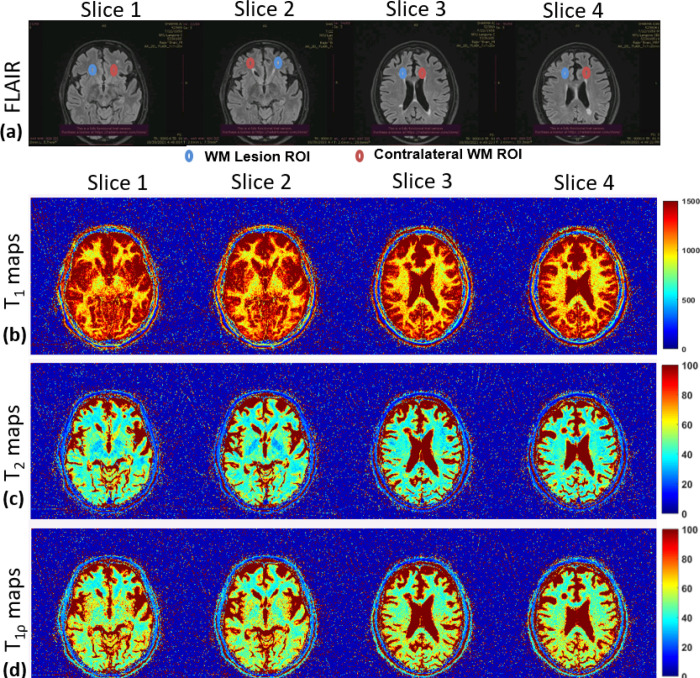
3D-MRF results in MS patient. (a) shows clinical imaging with FLAIR MRI four slices with identified lesions. Blue ROIs are drawn around the lesions, and contralateral ROIs are drawn in red. (b) to (d) show T_1_ maps, T_2_ maps and T_1ρ_ maps, respectively for each of the slices shown in (a)

The results of the 3D-MRF imaging in a representative MS patient is shown in figure 4. The top row (Figure 4(a)) shows standard non-contrast clinical FLAIR imaging in the MS patient. The 4 slices shown each contain lesions in the WM, and are highlighted in the images with the blue circles. Additional contralateral ROIs (red ROIs) were drawn on the opposite brain hemisphere from the lesion. Figure 4(b – d) show for each slice, the calculated T_1_ map, T_2_ map and T_1ρ_ map in the MS patient.

**Figure 5:**
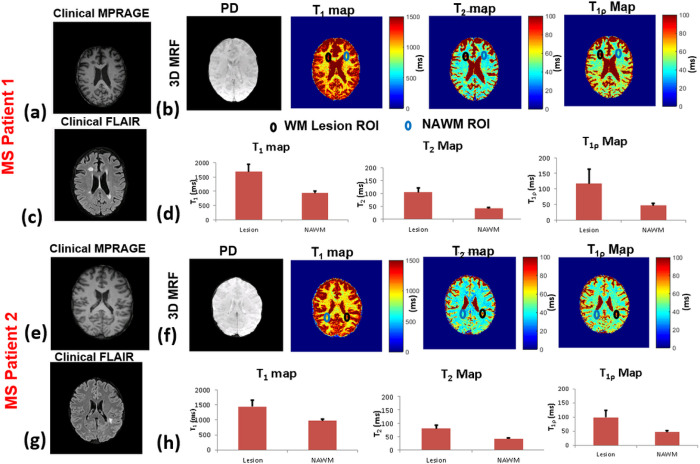
3D-MRF in two MS patients with chronic (a-d) and active (e-h) lesions. (a) non-contrast clinical T_1_ and FLAIR imaging in an MS patient with chronic lesions, (b) 3D-MRF maps with ROIs drawn in a WM lesion and contralateral normal appearing WM (NAWM). (c) comparison of mean values between WM lesion ROI and NAWM ROI from the T_1_-map, T_2_-map and T_1ρ_ maps. Sub-figures (d-f) show the results in a patient with enhancing lesions. The NAWM ROIs in both MS patients have similar values consistent with WM values, but the chronic MS lesion shows increased values for T_1_, T_2_ and T_1ρ_ compared to enhancing lesion ROIs suggesting the potential to discriminate chronic and active enhancing lesions using 3D-MRF.

Figure 5 shows the results of 3D-MRF imaging on two MS patients. Figure 5(a–d) shows results of an MS patient with chronic lesions (the same lesions were noted on MR scans for the past decade for this patient) and figure 5(e–h) shows results from an MS patient with actively enhancing lesions. Figure 5(a) and 5(c) shows T_1_ and T_2_ imaging using the standard non-contrast clinical imaging protocol. The T_2_ imaging shows the presence of MS lesions. Figure 5(b) shows 3D-MRF results showing PD, T_1_-, T_2_-, and T_1ρ_-maps. WM ROIs drawn in the WM lesion and contralateral NAWM showed significant differences in values for T_1_ (P=0.023) T_2_ (P=0.016) and T_1ρ_ (P=0.020) and are shown in the bar graphs in figure 5(c). Figure 5(e–h) shows the results from an MS patient with new enhancing lesions. Figure 5(e) and 5(h) shows standard clinical imaging that shows the presence of a lesion, and figure 5(f) shows the results from the 3D-MRF. The identified lesions are marked with black ROIs and the contralateral ROI is shown in blue. Comparison of T_1_, T_2_ and T_1ρ_ on the lesion side and the contralateral side shows significant differences. The bar graphs in Figure 5(h) show the significant differences between lesion ROIs and contralateral NAWM ROIs for T_1_ (P=0.006), T_2_ (P=0.020), and T_1ρ_ (0.016).

**Figure 6:**
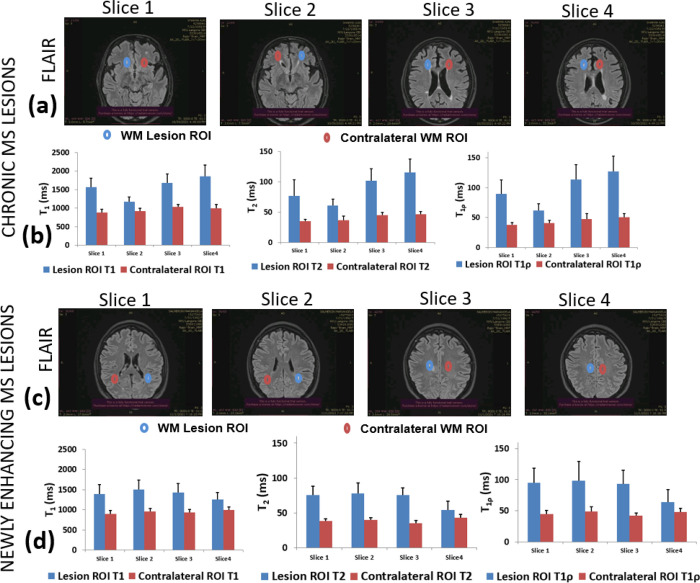
Comparison of Lesion ROI vs NAWM ROI in chronic and newly enhancing MS patients. (a) and (b) show results from chronic MS patient. (a) shows clinical FLAIR imaging with 4 slices with identified lesions. (b) shows mean values of T_1_, T_2_ and T_1ρ_ with SDs for each slice compared to their corresponding NAWM ROI. Sub-Figures (c) and (d) shows results from newly enhancing lesions. (c) shows clinical FLAIR imaging in an MS patient with newly enhancing MS lesions. (d) shows mean values of T_1_, T_2_ and T_1ρ_ with SDs for each slice compared to their corresponding NAWM ROI

Figure 6 shows more details of the MS patient with chronic lesions and the patient with newly enhancing lesions. In Figure 6(a), four slices are shown with clinical FLAIR imaging from the MS patient with chronic lesions. The ROIs drawn around the lesion are shown in blue and contralateral ROIs are shown in red. Figure 6(b) shows the comparison of T_1_, T_2_ and T_1ρ_ from each ROI and each slice from the MRF parameter maps. The bar graph shows significant differences in all parameters in each slice compared to the contralateral NAWM. Figure 6(c) shows similar FLAIR imaging from the patient with newly enhancing lesions. ROIs show the lesion and contralateral NAWM. Figure 6(d) shows significant differences in each slice for all the calculated parameters compared to the contralateral NAWM.

Table 3 shows the comparison of lesion ROIs, the contralateral NAWM ROI and control subjects ROI in similar brain locations as the lesion. For the MS patient with chronic lesions, the NAWM values compared to healthy controls are consistently and significantly higher (T_1_ (P=0.026), T_2_ (P=0.038), and T_1ρ_ (0.036)). For the MS patient with newly enhancing lesions, the NAWM values are trending higher for all parameters but are significantly higher for only for T_1ρ_ (T_1_ (P=0.07), T_2_ (P=0.48), and T_1ρ_ (P=0.012)). Supplementary information Table S1 shows the differences between chronic lesion ROI in Patient 1 (T_1_, T_2_, and T_1ρ_) vs the newly enhancing lesion ROIS in Patient 2 (T_1_, T_2_, and T_1ρ_). The quantitative parameter values for chronic lesions are clearly trending higher than the newly enhancing lesion ROIs. A detailed study investigating additional MS patients is needed to draw statistical inferences in MS patients.

## Discussion

In this study, we have demonstrated the use of MRF to acquire simultaneous 3D multi-parameter maps, quantifying T_1_, T_2_, T_1ρ_ in the brain, in a clinically feasible time and explored the possibility of extending it to MS applications for quantitative tissue characterization.

Multi-parameter mapping has been gaining popularity recently due to a number of advantages it offers ^17,25–27^. First, it allows to encode simultaneously multiple important parameters into a single imaging sequence. This allows the parameters to be automatically co-registered, and quantified simultaneously. Second, time and cost savings as a result of a single shorter scan. Individual mapping sequences are time consuming, and can take in excess of 40 min to complete. In comparison, the 3D-MRF implemented here can complete the acquisition in much less time (12 min for 2 shots). In a clinical setting, reduced time for scans will result in cost savings, and is desirable. Third, current diagnostic clinical scans and protocols for MS are not quantitative, and establishing quantitative metrics and better tissue characterization will give deeper insights into the mechanistic aspects of the disease at the clinical and sub-clinical level.

A number of recent developments allow quantification of T_1_, T_2_, T_2_*, diffusion^28^, perfusion and vascular parameters ^29^. A majority of the approaches using MRF are 2D multi-slice acquisitions. The 2D-MRF acquisition is susceptible to through plane motion, and takes longer to acquire. The 3D-MRF acquisition used here achieves better imaging efficiency, allows contiguous coverage, and eliminates through plane motion. Another approach called MR multi-tasking uses simultaneously acquired multi-parametric data using a low rank tensor model to generate parameter maps, has shown preliminary feasibility for MS imaging ^17^.

The estimated MRF parameters for T_1_, T_2_ and T_1ρ_ were within the expected range reported in literature ^2,12,20,30,31^. T_1ρ_ was consistently higher than reported T_2_ values with comparable ranges. The T_1_ values for WM and GM were in similar ranges compared to literature ^12,31^. There were some systematic biases however. There is some variability of values in repeatability studies from the Bland-Altman studies. This may be due to imaging sequence imperfections, or experimental variability. From the in vivo multi-shot data, two trends can be seen from going from data acquired with 1-shot to 4-shots. There is increased SNR and reduced variability among the ROIs in the brain. While the errors from single-shot data have more errors, the differences from 2-shot and 4-shot are small enough to use 2-shots for brain imaging, given the advantage of time-savings.

MRI has been the standard of care for diagnostic imaging in MS since 2001 ^32^. MS is a complex inflammatory, demyelinating disease affecting the central nervous system (CNS) ^32^. Diagnosis of MS requires evidence of disease progression in space and over time, as well as ruling out other disorders mimicking similar clinical profiles ^32,33^. Adding to this complexity in MS is the difficulty of classifying lesions and staging the disease in a spectrum ranging from active demyelination, mixed active and inactive demyelination to completely inactive demyelination stages ^34–36^. Furthermore, focusing only on visible focal lesions have shown to have poor correlations with disease progression ^34^, and diffuse sub-clinical effects are reported on NAWM and normal appearing gray matter (NAGM) too ^32,37^.

In this study, the NAWM values (for T_1_, T_2_, and T_1ρ_) in the chronic MS patient were significantly higher than control subject WM ROIs. Several previous studies have reported similar findings ^2,10,17,38^. The differences in NAWM values and controls may be due to a number of factors including axonal degeneration, inflammatory processes, myelin content reduction, and blood brain barrier leakage ^39^. A highlight of this study is the use of T_1ρ_ mapping in addition to T_1_ and T_2_ mapping. T_1ρ_ mapping on its own has been used for MS applications in a number of studies ^2,10,17^. T_1ρ_ as a contrast is sensitive to chemical exchange and lower frequency interactions and has been shown to be a sensitive indicator to lower frequency interactions between extra-cellular water and complex macromolecules ^40^. In this study we noted significant differences between T_1ρ_ values within the lesion and NAWM, as well as higher values in the NAWM compared to T_1ρ_ values in control subjects. T_1ρ_ may be able to detect GM demyelination too which is a more subtle change due low myelin content ^41,42^.

This study shows the results from two types of MS patients, a long-time diagnosed MS patient with many chronic lesions, and a newly diagnosed patient with recently enhancing lesions. The chronic MS lesions show increased values for T_1_, T_2_ and T_1ρ_ compared to newly enhancing lesions (Supplementary information Table S1), suggesting the potential to discriminate chronic and active enhancing lesions using 3D-MRF. In addition, the non-lesional NAWM ROIs in both MS patients have similarly elevated values compared to control subjects, consistent with literature ^2,10,35,38^. While the data presented here should not be used to make statistical conclusions, it suggests that quantitative characterization of MS patients (T_1_, T_2_, and T_1ρ_) may potentially be used to discriminate between lesions and possibly allow to stage MS patients. This could have important clinical implications in diagnosing and monitoring MS patients.

There are several future improvements that are possible to increase the coverage and resolution of 3D-MRF which will increase the utility and robustness in a clinical setting. The current 3D implementation is a stack-of-stars implementation, with Cartesian sampling in the kz-dimension, resulting in fold-over aliasing artifacts along the edge slices. Using a true 3D acquisition (along the kz dimension too) will eliminate this artifact, and result in improved usable coverage in the brain ^43^. Further improvements can be made to T_1ρ_ spin-lock durations, by optimizing the TSL durations using a method such Cramer-Rao lower bound (CRLB) ^44^ or improved sub-space reconstruction methods ^45^. This will reduce and optimize the number of TSL segments required and allow increased coverage. Finally, Deep Learning approaches to image reconstruction can replace the compute intensive offline reconstruction methods ^46,47^ to offer near real-time construction, which will have a big clinical impact.

This study has the following limitations. The major drawback is the coverage of the 3D-MRF for brain imaging. For MS applications, full brain coverage at high resolution is desired. While we achieve 1 mm in-plane resolution, more work is required to improve the through-plane resolution, and extend coverage to whole brain. This study shows a demonstration showing feasibility of quantitative 3D-MRF multi-parameter mapping in only two MS patients. Larger studies in MS applications with this technique are warranted to explore the feasibility in MS diagnosis and monitoring.

## Methods

### Study Design

This prospective study was approved by the New York University Grossman School of Medicine institutional review board (IRB), was health information portability and accountability act (HIPAA) compliant and all methods were performed in accordance with the IRB guidelines and regulations. All recruited MS patients and healthy volunteers provided written informed consent.

### MRF Sequence Design and Dictionary Construction

**Figure 7:**
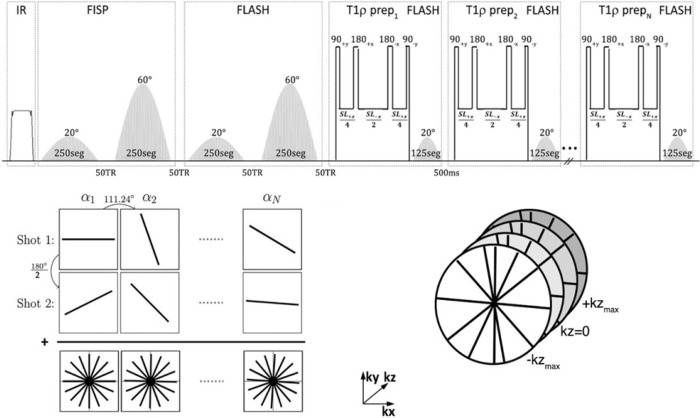
Pulse timing diagram of 3D-MRF sequence. The 3D-MRF sequence consists of an IR pulse, followed by a FISP module with two segments to encode for T_1_/T_2_, a FLASH module with two segments to encode for T_1_/B_1_, and a T_1ρ_ module to encode T_1ρ_. To increase SNR and k-space coverage, additional shots (n shots) were acquired by adding an offset angle (180°/n) at the beginning of each train. The 3D sequence uses readouts acquired in a center-out stack-of-stars in the kz dimension.

The 3D implementation of the MRF sequence is able to quantify four parameters: T_1_, T_2_, T_1ρ_ and B_1_+. The 3D-MRF sequence was based on a previous 2D implementation ^48^ and was extended to encode T_1ρ_, in addition to the original T_1_, T_2_ and B_1_ parameters for musculoskeletal applications ^24,49,50^. The 3D-MRF sequence timing diagram is shown in figure 7. A 10 ms adiabatic inversion pulse was used for inversion. This is followed by 3 modules. The first module consists of two FISP segments that encode for T_1_/T_2_, consisting of 250 slab-selective RF excitations. The first FISP segment has flip angle varying from 0° to 20° and the second FISP segment has flip angles varying from 0° to 60°. A 50 ms delay is used between the two segments to recover magnetization. The second module of the sequence is used to encode T_1_/B_1_+, consists of 2 FLASH segments. It uses similar RF excitations and FA variation as the FISP module. The third module encodes for T_1ρ_. A T_1ρ_ preparation pulse with variable duration (6 pulses with spin-lock duration varying from 2–45 ms) followed by 125 RF excitations for each spin lock pulse, with FAs ranging from 0° to 20°. Golden angle radial readouts following each RF excitation were used with center out readout in the kz dimension. At the end of the readouts for each spin-lock pulse, a 500 ms magnetization recovery delay is used. The T_1ρ_ preparation pulse uses balanced RF pulses to account for B_1_ inhomogeneities. It uses a hard 90_y_ pulse to flip the magnetization to the transverse plane. This is followed by 4 balanced alternating phase spin-lock pulses are applied. Two 1 ms, 180 hard refocusing pulses are used (180_+x_, 180_−x_) to account for B_0_ inhomogeneities.

To increase SNR and k-space coverage, additional data with additional shots (n shots) were acquired by adding an offset angle (180°/n) at the beginning of each train. The 3D-MRF imaging sequence (Figure 7) with 1 shot took 6 minutes, 2 shots took 12 min, 4 shots took 24 minutes, and 8 shots took 48 minutes. Other MR acquisition parameters included: FOV = 240 mm, in-plane voxel resolution= 1×1 mm^2^, 3 mm through plane slice thickness, TR = 7.5 ms, TE = 3.5 ms, bandwidth = 500 Hz/pixel, frequency of spin lock = 500 Hz.

All the algorithms were implemented in MATLAB (MathWorks, Natick, MA, USA). Extended phase graph simulations were performed to compute a dictionary of simulated MR fingerprints ^51^ with a T_1_ range of 50–3000 ms, T_2_ range of 2–200 ms, and a T_1ρ_ range of 2–200 ms in steps of 6%. The acquired data, as well as the simulated dictionary, were compressed using singular value decomposition (SVD) to speed up dictionary matching ^52^, which was performed offline. An iterative dictionary pattern matching algorithm was used to produce quantitative maps of proton density, T_1_, T_2_, T_1ρ_ and B_1_.

### Phantom Study

The 3D-MRF sequence was tested on a standardized ISMRM/NIST phantoms with published T_1_ and T_2_ values for 14 spheres ^53^. 3D-MRF data were obtained using 2, 4, 6 and 8 shots were obtained on a clinical MR scanner (Prisma 3T, Siemens Healthineers, Erlangen, Germany) using a vendor provided 20-channel receive only, birdcage head coil. Reference imaging sequences used for T_1_-mapping was a 3D spoiled gradient recalled echo sequence, for T_2_-mapping was a 3D spin echo sequence as described in the reference ^54^, and for T_1ρ_-mapping a custom 3D-T_1ρ_ mapping sequence ^4^ was used. The specific parameters used for each sequence compared are shown in table 1.

To compare the calculated parameters for the number of shots with the reference values regression and Bland-Altman analysis were performed for T_1_, T_2_ and T_1ρ_ values. Supplementary information figures S1, S2, and S3 show the results for T_1_, T_2_ and T_1ρ_, respectively.

### In-vivo Study

The study was approved by the institutional review board (IRB). To test the efficiency and errors in multiple shot acquisition schemes for neuro-imaging applications, we tested the 3D-MRF imaging on control subjects. Five control subjects (3 males, 2 females, 27±3 years) were recruited following informed consent, and in accordance with our IRB guidelines. They underwent brain imaging with the 3D-MRF imaging sequence at 1, 2 and 4 shots. To test repeatability, two subjects were scanned another time (~ a week later), and regression and Bland-Altman plots of the first scan and the repeat scan were performed. Intra-class correlation coefficients were calculated to quantify agreement between the scans. Manual regions of interest were drawn in the gray matter (GM) and white matter (WM) of the brain in all control subjects to quantify the variability of parameter estimation among the shots.

To test the 3D-MRF for MS applications, two MS patients (1 male, 63 years and 1 female, 29 years) with MS-diagnosed lesions were recruited following informed consent in order to assess the feasibility in detecting subtle pathological changes in normal appearing white matter (NAWM) and differentiating MS lesions. They underwent brain imaging with no contrast administration using the 3D-MRF sequence at 2 shots, followed by routine clinical imaging protocol without contrast of the whole brain consisting T_1_-MPRAGE for T_1_ contrast, and FLAIR imaging for T_2_ contrast. Manual ROIs were drawn around the identified WM lesions and contralateral hemispheres with NAWM.

### Statistical Analysis

For the phantom experiments, the mean and standard deviation of the ROIs drawn for each sphere across multiple slices was calculated. Regression and Bland-Altman analysis was performed to assess the agreement of multi-shot data with reference mapping techniques.

For the in-vivo control subjects, the mean and standard deviations in the manual ROIs drawn (The GM ROIs were drawn in the Insula and WM ROIs were drawn in the frontal lobe for all the subjects) across the subjects were calculated for 1, 2 and 4 shots. Test-retest reliability was assessed using regression plots, and Bland-Altman analysis. For each parameter, the intra-class correlation coefficient was calculated as :

$$ICC=\frac{\sigma_{b}^{2}}{\sigma_{b}^{2}+\sigma_{w}^{2}}$$

Where $\sigma_{b}^{2}$ and $\sigma_{w}^{2}$ are between subjects, and within subjects variation, respectively.

For the MS patients, manual ROIs drawn around WM lesions in the two patients were compared against contralateral sides and in ROIs drawn corresponding WM locations in the control subjects. Paired two-tailed Student T-tests were used to assess the difference in relaxation times between MS-lesion ROIs and NAWM or comparable WM regions in control subjects. A p-value of less than 0.05 was considered significant.

## Conclusion

In conclusion, this study demonstrated the use of a 3D-MRF technique that is able to quantify T_1_, T_2_ and T_1ρ_ for brain imaging applications in a clinically feasible time, and explored the feasibility of quantitative multi-parameter characterization in MS patients.

## Data Availability

The datasets used and/or analyzed during the current study are available from the corresponding author on reasonable request.
